# Effect of elevated oxygen concentration on bacteria, yeasts, and cells propagated for production of biological compounds

**DOI:** 10.1186/s12934-014-0181-5

**Published:** 2014-12-19

**Authors:** Antonino Baez, Joseph Shiloach

**Affiliations:** Biotechnology Core Laboratory, National Institute of Diabetes and Digestive and Kidney Diseases, National Institutes of Health, Bethesda, MD 20892 USA

**Keywords:** Hyperoxia, Oxidative stress, ROS, Protein oxidation, SOD activity, Hyperoxygenation

## Abstract

The response of bacteria, yeast, and mammalian and insects cells to oxidative stress is a topic that has been studied for many years. However, in most the reported studies, the oxidative stress was caused by challenging the organisms with H_2_O_2_ and redox-cycling drugs, but not by subjecting the cells to high concentrations of molecular oxygen. In this review we summarize available information about the effect of elevated oxygen concentrations on the physiology of microorganisms and cells at various culture conditions. In general, increased oxygen concentrations promote higher leakage of reactive oxygen species (superoxide and H_2_O_2_) from the respiratory chain affecting metalloenzymes and DNA that in turn cause impaired growth and elevated mutagenesis. To prevent the potential damage, the microorganisms and cells respond by activating antioxidant defenses and repair systems. This review described the factors that affect growth properties and metabolism at elevated oxygen concentrations that cells may be exposed to, in bioreactor sparged with oxygen enriched air which could affect the yield and quality of the recombinant proteins produced by high cell density schemes.

## Introduction

With the routine utilization of high density growth of microorganisms and mammalian cells for production of various biologicals, the use of oxygen-enriched air has become a common strategy. Elevated oxygen concentrations in the inlet gas may stress the cells in certain location in the bioreactor [1,2]. Although oxygen is essential to growth and production, it is also known to be toxic at elevated concentrations for a variety of cell types [3]. Most aerobic and microaerophilic organisms have developed protective responses to tolerate environmental oxygen concentrations. But when oxygen concentration surpasses the air saturation level, reactive oxygen species (ROS), such as superoxide (O_2_-) and hydrogen peroxide (H_2_O_2_), accumulate as byproducts of aerobic metabolism [4-6]. These molecules are toxic to the organisms since they are more reactive than molecular oxygen [7,8]. Most of the published research has focused on how these compounds are produced inside the cells; on how they damage the cellular components and on the defense mechanisms the cell activates to prevent the effect of these reactive species [8]. Yet, the damaging effect of molecular oxygen on the cell behavior has not been fully studied. The purpose of this review is to summarize the known information on the physiological response of organisms exposed to high molecular oxygen concentrations, especially the effect on growth, metabolism, enzyme activity, protein oxidation, and gene expression.

### Effects of high molecular oxygen concentration on growth and metabolism of prokaryotes

In the late 1960s, it was reported that oxygen concentrations above 100% air saturation cause inhibition of metabolism and respiration in microorganisms [9]. A few years later, the labs of O.R. Brown and I. Fridovich investigated the toxic effect of hyperbaric oxygen on *Escherichia coli* B and K-12 strains, *Streptococcus (Enterococcus) faecalis*, *Bacillus subtilis*, and *Saccharomyces cerevisiae* by exposing the cultures to 1, 4.2 and 20 atm pressure of O_2_ [10-13]. They showed that growth and respiration of *E. coli* grown in minimal salts medium was rapidly but reversibly inhibited by oxygen at partial pressures above 1 atm. The growth-inhibitory effect of hyperbaric oxygen was partially reduced by the addition of yeast extract to the culture medium and it was later established that 10 specific amino acids, niacin, and thiamin were necessary to maintain the cells growth capability at the elevated oxygen concentrations [14]. It was found that biosynthesis of branched-chain amino acids is inhibited by the high oxygen concentration [11,15]. But even with amino acid supplementation, the growth of *E. coli* AB1157 in glucose minimal medium was impaired when the cells were exposed to 1 atm of O_2_, and the growth was more affected when the tricarboxylic acid (TCA) cycle-dependent carbon source succinate was used [16]. Based on their observations, the authors concluded that aconitase is one of the enzymes that is affected by molecular oxygen. The inhibition of the tricarboxylic acid cycle is expected to cause a decrease in respiration as a result of limiting electron flow from the substrates to oxygen. The oxygen toxicity effect was more severe at the exponential growth phase than at the stationary phase [17].

The addition of 0.1 mM thiamine to *E. coli* K-12 culture was found to protect the cells from the inhibitory effect of hyperoxygenation (4.2 atm of O_2_) that caused 3-fold decrease in the intracellular thiamin concentration [14]. Thiamin diphosphate is a cofactor involved in the pentose phosphate pathway and hence may affect NADPH biosynthesis. The synthesis and intracellular concentration of the pyridine nucleotide coenzymes were studied and found to be lower in oxygen-poisoned cells [18-21]. These findings contributed to the use of hyperoxygenation for medical applications. Hyperbaric oxygen has been used for the treatment of serious infections of *Staphylococcus* and *Vibrio vulnificus*; 90 min exposure to 2 atm of O_2_ significantly inhibited growth of *Staphylococcus* resistant strains in patients [22,23].

The impact of dissolved-oxygen (dO_2_) concentration on *E. coli* processes for production of recombinant proteins is uncertain [24,25]. Increasing dO_2_ concentration from 50% to 100% air saturation (0.105 to 0.21 atm of O_2_) did not affect the specific activity and the volumetric productivity of the expressed recombinant β-galactosidase, nor the growth rate, biomass concentration, or plasmid content of *E. coli* TB-1/pUC19 and TB-1/p1034 [24]. However, the usage of oxygen enriched air (0.40 atm of O_2_) to control dO_2_ concentration above 20% air-saturation in a glucose defined medium contributed to higher biomass and higher acetate concentrations but decreased the specific production of rhGH produced by *E. coli* K-12 strain compared with air supply control experiment [25]. In contrast, the productivity of rhGH in *E. coli* BL21 growing in a complex medium and using 0.93 atm of O_2_ in inlet gas to control dO_2_ at the same levels (20% air saturation), was 2-fold higher than in air-control culture [26]. In addition to the difference in the *E. coli* strain and induction time, the availability of amino acids in the complex medium might explain the contrasting results. By using a higher oxygen concentration (0.68 atm of O_2_) in the inlet gas to maintain a dO_2_ of 30% air-saturation in a glucose defined medium, Lara et al. [27] did not observe any acetate or other organic acid accumulation. The authors concluded that any potential oxidative stress due to the oxygen content in the gas anywhere in the bioreactor was negligible since no changes on overflow metabolism such as acetate excretion was observed.

Longer exposure time to elevated oxygen concentrations and stronger oxidative stress might occur in large bioreactors when gas blending or pure oxygen is used for scaling-up *E. coli* cultivations [1,27]. Potential physiological effects of the use of oxygen enriched air were studied by Baez and Shiloach [28]. No significant effects on the growth rate, biomass yield, and maximum acetate accumulation was found when *E. coli* batch cultures were exposed to dO_2_ of 300% air-saturation (~0.63 atm of O_2_ inlet gas). However, oxygen uptake and acetate production rates decreased when oxygen concentration was increased in chemostat cultures [28]. An increasing maintenance coefficient was suggested by Castan et al. [25] as a result of the respiration changes caused by high oxygen; this observation is in line with the maintenance coefficient of *E. coli* MG1655 growing in glucose mineral medium that was found to increase from 0.66 to 0.85 mmol/g-h following increase in oxygen concentration from 30% to 300% air saturation (Baez, unpublished data).

### Effects of elevated molecular oxygen concentration on growth and metabolism of eukaryotes

Unlike prokaryotes, eukaryotes are more sensitive to atmospheric oxygen concentration above 40% [29,30]. Cultures of human myeloid leukemia U-937 cells exposed to 50% of O_2_ (~240% air saturation) showed alteration of the mitochondrial respiratory chain, lactate and alanine accumulation, and strong growth inhibition compared with cells exposed to 21% O_2_ [31]. Exposing Chinese hamster ovary (CHO) cells to 80% O_2_ (~380% air saturation) inhibited cell growth together with 65% higher glutathione accumulation compared with cultures grown at 20% O_2_ [30]. Specific growth rate, production productivity of erythropoietin (EPO), and final cell yield of CHO cell transfected with the human EPO gene was significantly lower at 200% air saturation, but the cells were unaffected by oxygen concentrations between 10% and 100% air saturation [32]. Hyperoxic conditions (200% air saturation) also caused lower fucosylation pattern in the produced EPO. On the other hand, moderate oxygenation of CHO cell culture (80% air saturation) did not affect viability, productivity, and cell-specific glutathione production rate compared with a control culture exposed to 30% air saturation [33,34]. When mouse-mouse hybridoma cells were exposed to 200%, 300% and 476% air saturation, the cells were not able to grow and an association between DNA strand breakage and hyperoxia was observed compared with a control culture growing at 10% air saturation [35]. Viral infection of mammalian and insect cells is associated with increased oxidative stress [36,37]. The increase in dissolved oxygen (dO_2_) concentrations decreased the viability and increased the protein carbonyl content and lipid oxidation of infected Tn-5B1-4 cells, but did not affect the viability, protein and lipid oxidation of Sf-9 cells [38,39]. When, mouse lung epithelial type II cells (MLE-12) were exposed to hyperoxia (95% O_2_) they became larger and showed significant decrease in basal oxygen consumption rate, glycolytic capacity and growth rate compared to cultures under normoxia (room air) [40]. Since elevated O_2_ concentrations inhibit complex I and II activities and decrease the oxygen consumption rate via complex I and II, but not complex IV, in isolated mitochondria, it was concluded that complexes I and II are specific targets of hyperoxia [40,41]. *S. cerevisiae* mutants devoid of mitochondrial superoxide dismutase activity were more vulnerable to hyperoxia (100% O_2_) than *S. cerevisiae* lacking cytosolic superoxide dismutase activity, suggesting that the mitochondrial respiratory chain is the target of hyperoxia toxicity [42].

### Molecular responses to elevated oxygen concentrations

#### Accumulation of reactive oxygen species (ROS), and effect of oxygen on protein and lipid oxidation

**Figure 1:**
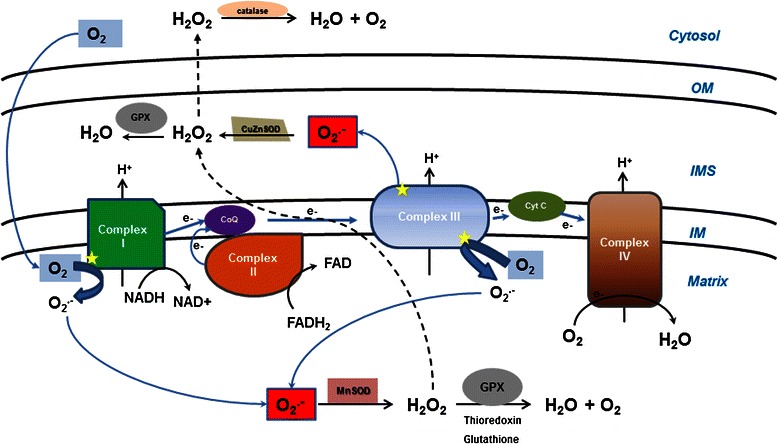
**Elevated oxygen triggers intracellular ROS accumulation.** The ubisemiquinone intermediates present in complexes I and III of the respiratory chain are the primary source of univalent reduction of oxygen into superoxide (O_2_^.-^) (indicated by stars). At over-oxygenation conditions, electrons leak from complexes I and III generating superoxide increases. Superoxide is converted immediately to hydrogen peroxide (H_2_O_2_) by manganese superoxide dismutase (MnSOD) or copper/zinc superoxide dismutase (Cu/ZnSOD). At normoxic conditions, the catalases and peroxidase systems minimize the accumulation of H_2_O_2_ but at higher oxygen concentrations; these antioxidant defenses can be overwhelmed and the accumulating H_2_O_2_ diffuse freely from the mitochondria (dashed lines) reaching targets that can be damaged such as dehydratases and DNA. Abbreviations: CoQ, coenzyme Q10; Cyt C, cytochrome C; GPX, glutathione peroxidase; IM, inner membrane; IMS, intermembrane space; OM, outer membrane.

When cells are exposed to high extracellular oxygen concentration, oxygen diffuses through the membranes and abstract electrons from reduced flavoenzymes to produce partially reduced oxygen species such as superoxide (O_2_^-^) and hydrogen peroxide (H_2_O_2_) [5,8,43,44]. Since ROS production-rate is proportional to collision frequency of oxygen and redox enzymes, the rate of O_2_- and H_2_O_2_ formation inside the cells depends directly on the oxygen concentration in the extracellular environment [4,5,44,45]. The association between hyperoxia and accumulation of ROS was shown in 1982 by Crapo and colleagues [46,47]. In later publications, it was shown that cultures of CD14+ monocyte and HeLa-20 cells exposed to 40% O_2_ and 80% O_2_ respectively produced 2-fold higher ROS amounts than cultures under normal atmospheric oxygen concentration [41,48]. Similarly, *Entamoeba histolytica* and *Drosophila melanogaster* flies exposed to high-oxygen environment (90-95% O_2_) showed 2-fold increases in ROS accumulation compared with flies exposed to normal oxygen conditions [49,50]. Using isolated mitochondria, it was shown that the majority of ROS detected in the cells were derived from the mitochondrial electron transport chain [41]. This was also established in bacteria, where the main source of endogenous superoxide (O_2_^-^) was found to be the respiratory chain [4,51]. It was also demonstrated that the formation rate of O_2_^-^ increase in proportion to the oxygen concentration [4]. Hence, it was proposed that at hyperoxia conditions, the main ROS accumulated in the mitochondrial matrix is H_2_O_2_. The proposed steps for its accumulation are the following: when cells are exposed to an increasing oxygen concentration, there is higher leakage of electrons from complex I and III of the respiratory chain leading to an increase in superoxide production as described in Figure 1. This superoxide is immediately converted to H_2_O_2_ by the mitochondrial superoxide dismutase [42]. At lower oxygen concentrations, catalases and glutathione peroxidase systems minimize the accumulation of H_2_O_2_ [44], but at higher oxygen concentrations, these antioxidant defenses are overwhelmed resulting in accumulation of H_2_O_2_ which can diffuse freely from the mitochondria reaching targets that can be damaged such as dehydratases and DNA [5,8].

Similarly two transcription activators (Yap1 and Skn7) involved in the oxidative stress response and redox homeostasis that are normally induced by H_2_O_2_, were also activated at elevated oxygen conditions. These suggested that elevated oxygen promotes H_2_O_2_ accumulation which in turn activated the transcription of Yap1 and Skn7 factors [42,52].

The increased generation of ROS during exposure to high-oxygen concentrations can overwhelm the antioxidant defense mechanisms and eventually cause cell death [53]. It is interesting to note here that hyperoxia did not increase the ROS content in oxygen-tolerant HeLa-80 cells or in oxygen-resistant *Drosophila melanogaster* flies as it did in the non-resistant controls [41,50]. Compared with the wild Type HeLa 20, the oxygen-tolerant HeLa-80 cell did not show significant differences in antioxidant enzymes or compounds nor differential susceptibility to apoptotic death; the only difference was the lower intracellular ROS accumulation by the oxygen-tolerant cell line [41]. The oxygen-tolerant flies showed similar antioxidant enzyme activity as the control flies but demonstrated lower H_2_O_2_ accumulation, lipid peroxidation and protein oxidation [50]. Based on the above, it was concluded that oxygen-tolerant cells do not rely on increased activity of antioxidant defenses but on an increased efficiency of mitochondrial metabolism which decreased ROS leakage from the electron-rich intermediates of the mitochondrial electron transport chain.

High oxygen concentrations also induced ROS accumulation in bacteria; it was found that the production of periplasmic superoxide increased proportionally to the oxygen concentration [4]. Superoxide released by the respiratory chain in the periplasm provoked glutathione efflux from the cells. In the absence of SodC activity, glutathione can catalyze the dismutation of superoxide into H_2_O_2_, protecting the cell from oxidative stress [54]. *E. coli* grown at 300% dO_2_ air saturation (~0.63 atm of O_2_) accumulated 22% higher intracellular ROS compared to cultures grown at 30% dO_2_ [28]. However, this ROS increase did not cause any detectable change in the oxidation pattern of proteins after 3 hours of induction at 300% dO_2_ [55]. In another example the carbonyl content of the total proteins of *Lactobacillus sake* increased 2.2 and 11.2 fold in insensitive and sensitive strains respectively after 72 hours of exposure to 0.9 atm of O_2_ [56]. The increased protein damage reported may have resulted from longer exposure to oxidative stress imposed by elevated O_2_. It is important to emphasize that when oxygen concentration in bacterial cultures is increased 10-fold, the intracellular ROS concentration is increased only 1.2 fold, while smaller oxygen increase (2 to 5 fold) in eukaryotes promote 2-fold higher intracellular ROS accumulation [28,41,48-50].

#### Inducible responses and enzyme inactivation

The physiological response of microorganisms to oxygen has been studied by comparing normoxic (room air) with anaerobic conditions. However, physiological response to increasing oxygen concentrations from normoxia to hyperoxia is also important since a set of protecting genes involved in redox homeostasis and oxidative stress are also activated. An example is the 36 fold activation of sulfiredoxin (Srx1) transcription and 12 fold activation of NADPH dehydrogenase (Oye3) transcription in *S. cerevisiae* exposed to hyperoxic conditions [42]. *E. coli* MG1655 growing in glucose minimal medium or *E. coli* AB1157 growing in complex medium activated the transcription of SoxRS-controlled genes in response to oxygen increase unlike genes under OxyR control [28,55]. Using *sodA-lacZ* and *sodB-lacZ* fusions, it was shown that oxygen activates *sodA* expression while it has an opposite effect on *sodB* expression [57]. *sodA* expression at 0.21 atm and 0.6 atm of O_2_ was 35 and 45 fold higher compared with anaerobic conditions. At the same time, 0.21 atm of O_2_ repressed *sodB* expression by two fold compared with the expression at anaerobic conditions. When exposed to increased oxygen, the double mutant *E. coli* strain sodA^-^ sodB^-^ activated the transcription of the genes under control of both the OxyR and SoxRS regulons [28]. In addition to the 32-fold up-regulation of *soxS* gene, two oxidant-resistant isozymes, aconitase A (*acnA*) and fumarase C (*fumC*), were up-regulated in the sodA^-^ sodB^-^ mutant, an indication that the unstable aconitase B and fumarases A and B were inactivated by the oxygen-shift [28]. The inactivation of these enzymes could be attributed to the accumulation of intracellular superoxide [58]. This is probable since most dehydratases containing the [4Fe-4S]^2+^ cluster and metalloenzymes in general, can be easily inactivated by superoxide generated through hyperoxygenation and redox-cycling agents [21,58-61]. In *Pseudomonas putida* KT2440, elevated oxygen was found to activate gene expression of the iron-sulfur cluster system while decreasing intracellular levels of free iron in the cells [62].

*E. coli* contains at least three distinct superoxide dismutases (SODs): Mn-containing SOD (gene product of *sodA*) and Fe-containing SOD (gene product of *sodB*), both localized in the cytoplasm, and the hybrid CuZn-containing SOD (gene product of *sodC*) which is located in the periplasmic space [63-65]. It has been known that these SODs enzymes play an essential role in protecting the bacteria from oxygen poisoning. Exposing *E. coli* or *S. cerevisiae* cells to 1 atm of O_2_ after growing at air atmosphere induce an increase in the SOD activity which allows the cells to resist the lethal effect of 20 atm of O_2_ [12,66]. However, microorganisms that were unable to respond to increase O_2_ concentration by increasing SOD activity, like *B. subtilis*, were sensitive to hyperbaric dissolved oxygen even when the catalase activity was increased [66]. Construction of mutant strains lacking these scavenging enzymes has confirmed the important role of SODs at elevated oxygen concentrations. Hence, *S. cerevisiae* mutants with compromised SOD activity were extremely sensitive to hyperoxia [42,67,68]. Similarly, *E. coli* strains lacking Mn-SOD and Fe-SOD were sensitive to elevated oxygen concentrations and showed higher rate of spontaneous mutagenesis than the parental strains [28,69]. This double mutant *E. coli* strain, as a result of being auxotrophic for branched-chain amino acids, was unable to grow in aerobic minimal-medium [70,71]. Additional example of the important role of SOD at elevated oxygen concentration is the behavior of *Lactobacillus sake*, the lower SOD activity in the sensitive strain causes higher superoxide accumulation and higher damage of the the [Fe-S]-cluster-containing enzymes [56]. All the above demonstrates that one of the critical roles of SOD is maintaining the stability of [4Fe-4S] cluster of dihydroxyacid dehydratase, fumarase A and B, promoting the aerobic growth [72,73].

## Conclusions

The use of oxygen enriched air has become an accepted practice currently utilized to support the aerobic growth of both prokaryotic and eukaryotic cells in bioreactors in an effort to increase the cell density and to improve process productivity. However, exposing cells to high oxygen concentrations is known to enhance the accumulation of intracellular ROS which can reach harmful levels that can overwhelm the antioxidant defenses and repair systems of the cells. The observations summarized here indicate that bacteria are more resistant to hyperoxygenation than mammalian cells. The ability of bacteria to control tightly the ROS homeostasis at elevated oxygen concentrations allows them to survive better in hyperoxia. The addition of agents that reduce the leak of electrons from respiratory chain hence reduce ROS accumulation, may prevent the damage of elevated oxygen concentration.
